# A workflow and protocol describing the field to digitization process for new project‐based fossil leaf collections

**DOI:** 10.1002/aps3.1025

**Published:** 2018-03-07

**Authors:** Dori L. Contreras

**Affiliations:** ^1^ University of California Museum of Paleontology 1101 Valley Life Sciences Building Berkeley California 94720 USA

**Keywords:** curation, digitization, fossil leaf collections, imaging, paleobotany, workflow

## Abstract

**Premise of the Study:**

This article provides a workflow and protocol for paleobotanical researchers that integrates project‐based fossil leaf specimen and data collection with curation and digitization. The methods aim to facilitate efficient digitization of new collections by researchers during the course of their study and promote public databasing of new specimen and project data.

**Methods and Results:**

The workflow was developed and refined to facilitate a project reconstructing an extensive fossil forest from leaf impressions/compressions. The workflow consists of field, museum, and data mobilization components. Customizing a workspace and streamlining all steps of specimen data collection, curation, and digitization into an integrated processing pipeline resulted in faster accumulation of specimen data and images.

**Conclusions:**

These protocols provide paleobotanists with logistics‐focused methods for integrating research with digitization, and are particularly applicable at institutions with limited collection support staff or when specimen images are needed for project purposes.

Over the past decade, the digitization and sharing of the vast biodiversity data contained in natural history collections has become a priority of natural history institutions and museums worldwide, and is viewed as a crucial step toward maximizing the scientific and societal potential of collections (Baird, 2010; Scoble, 2010; Beaman and Cellinese, 2012; Page et al., 2015). The processes involved in digitization, particularly imaging of individual specimens, can be costly in terms of person‐hours and grant funding, especially if carried out after the original research and curation (Vollmar et al., 2010). This poses logistical challenges for smaller museums or academic institutions with limited staff or funding dedicated to various curatorial and digitization tasks. For new project‐based collections, however, efficient workflows can enable researchers to complete these tasks during the course of their study by integrating project‐based data collection with curation and digitization (as recommended by Berents et al., 2010). When appropriate, researcher‐driven processing can increase time and cost efficiency of digitizing new collections while simultaneously supporting project goals. It entails immediate cataloging and imaging of newly acquired specimens without the need for additional personnel, adds the potential to use specimen images for data collection (e.g., digital measurements), and facilitates the sharing of digitized collections and project data immediately following completion and publication of research.

Currently in paleobotany, there is a general lack of published workflows for researchers that extend from the collection of new specimens and data in the field to the curation and digitization of specimens and associated data in a museum. The characterization of fossil plant communities and landscapes from leaf macrofossils typically requires large sample sizes and involves the description of numerous taxa new to science, thus generating extensive collections of specimens that are challenging and time‐consuming to manage and process (e.g., Iglesias et al., 2007; Wing et al., 2012). Although methods for collecting and characterizing fossil leaf floras are well described in the paleobotanical literature (e.g., Johnson, 2002; Peppe et al., 2008; Wing et al., 2012), they are generally geared toward sampling design and data collection and do not address protocols for specimen processing in the museum. Conversely, applicable digitization workflows are mostly concerned with mass digitization of pre‐existing collections (e.g., Nelson et al., 2012; Karim et al., 2016) and exclude field collection of specimens and research activities. The publication of workflows incorporating all phases of a research project, curation, and digitization will be useful for new researchers and students working on new fossil leaf floras, particularly at institutions without established protocols or that have limited curatorial support.

This article presents a workflow and protocol developed for the reconstruction of a diverse fossil flora from a laterally extensive deposit. The generalized workflow (Fig. 1) outlines the major components of the researcher‐driven process, extending from collection of specimens and data in the field through the transfer of digitized data to public online databases. The protocol (Appendix 1) details specific methods for community analysis of fossil leaf macrofloras within the framework of the workflow, focusing on the logistics of integrating research and digitization tasks for new collections. Although the protocol (Appendix 1) is specific to fossil leaf floras, the components of the researcher‐driven workflow (Fig. 1) are generalized enough that they can be adapted for other types of specimen‐based research.

**Figure 1:**
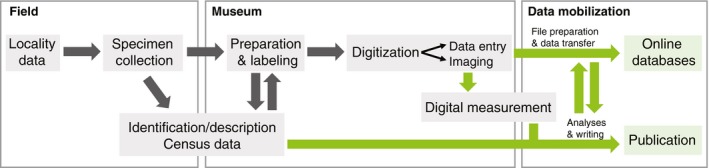
Generalized workflow for new project‐based fossil collections, extending from fieldwork through digitization and online databasing. The workflow is divided into three main components: field, museum, and data mobilization. Major steps in the workflow are depicted in solid gray boxes, organized in a linear fashion consistent with the project timeline progressing from left to right. Gray arrows depict the flow of processes involving physical specimens and data, whereas green arrows depict the flow of digital data. Detailed explanations of processes are provided in the text and Appendix 1.

## METHODS AND RESULTS

The protocol (Appendix 1) and workflow (Fig. 1) presented here are the result of four years of developing, testing, and refining field and museum methods for a single large‐scale paleobotanical project. The project aims to reconstruct the community structure and composition of a Cretaceous forest preserved in an extensive recrystallized volcanic ashfall deposit. The reconstruction is based on fossil leaves preserved as impressions and compressions, which were studied and collected on private land. Description of the plant community includes the following types of data, all of which are accommodated within the protocol: (1) species diversity (as fossil leaf morphotypes [Johnson, 1989; Ellis et al., 2009]), (2) relative abundance and percent cover of morphotypes and clades (as, respectively, number of fossil leaf specimens and number of 2‐cm increments following the line‐intercept method of Wing et al. [1993, 2012]), (3) spatial patterns of species composition across the deposit, and (4) functional diversity measured from leaf traits (e.g., Royer et al., 2007). These measures require a spatially explicit, quantitative sampling scheme and large sample sizes. Therefore, field censuses of leaf specimens were conducted at multiple discrete sampling sites across the deposit (over 20 quarries spanning the >1.2 km exposure). Field censuses entail onsite identification and quantification of fossil leaf specimens and enable a greater number of specimens to be sampled than can reasonably be collected and stored (see Johnson, 2002; Wing et al., 2012). In order to perform identifications during field censuses, the diversity of leaf morphotypes was first determined from an initial set of collections. These included samples from the first 10 quarries spanning the exposure, specimens from selective collecting of the deposit and float, and collections made in the 1990s and 2012 housed at Texas State University San Marcos (Upchurch and Mack, 1998). After circumscribing leaf morphotypes, descriptions and images of each were compiled into a morphotype guidebook that was used to identify specimens in the field. The guidebook was expanded upon as additional morphotypes were discovered during censusing. Overall, more than 2000 fossil specimens were collected during the initial diversity sampling and later census surveys and brought to the University of California Museum of Paleontology. These include type/voucher specimens for morphotypes, well‐preserved specimens for trait measurements, and specimens needing more detailed study at the museum. The general workflow (Fig. 1) and protocols (Appendix 1) were developed as a means to most efficiently streamline specimen‐based data collection in the field and museum with curation and digitization of the collected specimens.

The workflow has three major components: the field component, the museum component, and data mobilization. The following paragraphs outline these components as presented in Fig. 1 and summarize the steps and rationale of the corresponding paleobotanical protocol (see Appendix 1). These protocols are meant to be carried out by a single researcher or small research team (e.g., primary investigator and student apprentices), and are designed for specimens that are relatively two‐dimensional and can be studied and imaged without histological or three‐dimensional techniques, such as computed tomography scanning. Because these protocols were developed based on collections made on private land, they do not address the permitting, sampling, reporting, and legal issues associated with collecting on public or international lands. It should be noted, however, that all applicable laws and protocols for a particular area of study should be followed. Furthermore, the overall purpose of these protocols is to address logistical aspects of integrating specimen and data collection with curation and digitization, rather than to prescribe data analyses or specific sampling schemes for reconstruction of fossil plant communities. Customization of these protocols is expected for use at other institutions and for other projects.

### Field workflow

The field component of the workflow can be generalized as consisting of locality data collection, specimen collection, specimen identification/description, and census counts (Fig. 1). Field protocols are designed for taking multiple independent samples across a deposit and include two phases: making collections for an initial diversity survey, followed by quantitative field censuses of specimens at each sample site (Appendix 1, steps 1–4). These phases are completed at different times, each followed by processing in the museum. After establishing an initial set of sampling localities across the deposit, small collections are made from each and brought back to the museum to study and delineate taxa. These samples provide a working baseline for leaf diversity and are used to compile a field morphotype guidebook to be used and expanded during subsequent censuses. Field censuses of leaf specimens at each locality are performed to increase sample sizes for community description. The field protocols are designed to retain high‐precision locality information (e.g., GPS coordinates, depositional context, sediment samples) and field‐generated data (e.g., fossil identifications and census counts) with each individual fossil that is collected. This is primarily accomplished by assigning unique identification numbers to the rocks while in the field and treating each small sampling site (e.g., quarry) as a separate locality. After exposing the fossils (Appendix 1, step 2), each slab of rock is labeled with the locality number and a unique “Rock ID” number that is used for recording and maintaining field data (Appendix 1, step 3). During censuses, specimens on each slab are identified using the morphotype guidebook and all count and other census data are recorded by the Rock ID number (Appendix 1, step 3). Because of the limitations of collecting and storing all specimens from censuses and the lack of necessity for doing so, specimens are only collected if they: (1) may be used as vouchers or type specimens representing unique species or morphotypes, (2) have exceptional preservation that can be used for other data collection (e.g., trait measurements), or (3) could not be identified in the field but are of sufficient preservation to identify with further study (Appendix 1, step 4). Thus, the fossils collected represent a subset of those identified and censused in the field, as well as additional fossils that need to be identified and/or described.

### Museum workflow

The museum portion of the workflow consists of the transition phase and the processing pipeline. The transition phase (Appendix 1, steps 5–7) of the protocol organizes the newly collected fossils for efficient processing and connects field‐generated data with the specimens. The processing pipeline integrates identification and description of specimens, as well as any other data collection (e.g., census counts), with curatorial tasks and digitization. This integrated processing pipeline (Appendix 1, steps 8–14) allows for a single drawer of specimens to be pulled and all specimens processed to completion before moving to the next. The fundamental components of the pipeline are illustrated in Fig. 1, with more detailed explanation for the paleobotanical protocol in Appendix 1 (steps 8–14). The overall process is intended to be linear; however, some aspects of data collection and preparation can be iterative, as shown by reciprocal arrows in Fig. 1, and can vary based on the needs of a given specimen. During the transition phase, fossils are unpacked in the museum (Appendix 1, step 5) and grouped first by locality and then by morphotype or “like” specimens within drawers. Although this process can be time‐consuming, having similar specimens grouped together helps with any identifications or descriptive work needed during processing. The Rock IDs are recorded into a curatorial log and reconciled with the field census data (Appendix 1, step 6). This bridges data from the field to museum and allows easy determination of which rocks have already been identified and censused, and which need further work. The processing pipeline then proceeds as follows for each drawer. Rocks can be processed individually or in small groups of similar specimens as needed for identifying/delineating taxa. Specimens on each rock are prepared by removing any overlying rock matrix to expose each specimen as completely as possible (Appendix 1, step 8). If a rock was censused in the field, specimens are identified using the census data record. Otherwise, each specimen (or group of “like” specimens) is studied and then identified using the morphotype guidebook and comparison with morphotype voucher specimens. When necessary, specimens are described as a new morphotype (Appendix 1, step 9). Pre‐existing morphotype descriptions are also updated at this time if the newly collected specimens provide additional information or clarification of features. If the rock was not censused completely in the field, census counts are performed or updated (Appendix 1, step 10). Individual specimens are then assigned unique catalog numbers and labeled individually on the rock surface, according to museum protocols (Appendix 1, step 11). Once provided unique numbers, specimens can move to the digitization process, which includes entry into the collection database and imaging (Fig. 1). Although the pipeline is designed to accommodate cataloging and imaging of all individual specimens, this may not always be feasible or necessary and will depend on institutional protocols. In general, specimens should be imaged following a predetermined imaging plan (Karim et al., 2016), whether it be for all specimens or only select specimens, such as well‐preserved and/or potential type specimens (Appendix 1, step 12). Imaging of specimens should also account for project needs, such as for generating figures for the morphotype guidebook or for digital measurements of specimen traits (Appendix 1, step 15). Basic image processing, such as lighting adjustments and the addition of metadata to files, can be completed at the time of imaging if being applied to individual photos. Specimen data are then entered into the museum's digital collection database according to museum protocols (Appendix 1, step 13). Upon completion of the pipeline, each rock can be returned to the original drawer, or placed in a new one (e.g., with type specimens or grouped by morphotype) if desired, for the final organization of the collection (Appendix 1, step 14).

A key aspect to successful implementation of the integrated processing pipeline is the customization of a workstation that contains all, or most, of the equipment necessary for data collection, curation, and digitization (Appendix 1, step 7). The basic set‐up of the workstation comprises a workbench (e.g., two 6‐ft tables) lined with small task stations for fossil preparation and microscopy, labeling, photography, and data entry. The fossil preparation station is intended to accommodate small‐scale, precision preparation work and can consist of tools such as needles, picks, and small chisels with a jewelry hammer. More substantive preparation involving equipment that is large or subject to safety requirements (e.g., airscribes, mechanical splitters) may need to be completed in other designated locations as determined by the institution's facilities. Although purchase of new equipment for the workstation can be cost‐prohibitive, this can sometimes be mitigated by opportunistically acquiring second‐hand, often older, equipment from various other laboratories and/or moving equipment from other spaces of the museum. In some cases, the expenditures and supplies required for curation and digitization can be obtained by including them as expenses in grant applications for the research project. An integrated, centralized workstation provides several benefits, including reducing movement of specimens between facilities, improved flow between tasks that are often iterative (see Fig. 1), and sustained focus on each specimen through its completion. Furthermore, having all task stations centralized facilitates small teams of people working together simultaneously on a set of fossils. Student research apprentices and volunteers can be instrumental in processing large amounts of specimens quickly. The integrated workstation aids in the management of students and volunteers and improves quality control by allowing constant, open communication about specimens as they are moving through the pipeline. Recruitment of students at academic institutions can also benefit from the integrated research‐curation process, because students are able to participate directly in research and get a more comprehensive experience than when participating solely in data collection or curatorial activities.

### Data mobilization

The final component of the workflow is the mobilization of digitized specimen records, research results, and data sets (Fig. 1; Appendix 1, step 16). The overall goal is timely and efficient digital sharing and archiving of both specimen and project data after completion and publication of the research. The specific trajectories of different types of data will vary based on institutional practices regarding databasing, the platforms available for different data types, and project specifics, including plans for further research on the specimens. Therefore, a thorough digital data management plan should be devised in advance that identifies all of the types of data that will be produced throughout the study (e.g., specimen images, museum specimen records, community abundance data, species occurrence data, trait data) and where the data will eventually be stored. Any researcher‐generated data or project‐management files should be customized so that file types, data entry fields, formatting, and organization are tailored to the intended final platform from the outset of the project. This will reduce the amount of post‐processing and file preparation necessary to upload data to online databases at completion of the study. Suggestions for file formatting and online databases for each of the data files generated by the paleobotanical protocol are provided (Appendix 1, see *Data files).

### Overall methodology

For researcher‐based processing of a large collection (up to ~2000 specimens), the integrated processing pipeline was found to be more efficient than processing all specimens, or large batches of specimens, on a single‐task basis. Large‐batch processing was the first method attempted, primarily as a result of various equipment (e.g., microscopes vs. photography equipment) being located in different rooms or facilities that are separate from each other and the bulk of the collections. The batch processing was both time and space inefficient because specimens were handled on multiple separate occasions, carted to different workspaces to complete tasks, and accumulated on workbenches before processing batches of specimens for a single task. This created bottlenecks in the flow of specimens and data. Iterative processes also resulted in additional handling of specimens and shifting between facilities that slowed progress. The integrated processing pipeline, however, results in a continuous flow of data and completed specimens. Key timesaving aspects of the overall workflow include the efficient transfer of field‐collected specimen data to the museum, the streamlining of processes into an integrated processing pipeline, organizing a workspace to accommodate all tasks in one space, and involving undergraduate students and volunteers in the project for more effective team processing of large amounts of material.

## CONCLUSIONS

The protocols and workflow presented here are directly applicable for paleobotanists interested in the quantitative reconstruction of fossil leaf macrofloras. The general principles of the workflow—integrating specimen‐based research with curation and digitization—apply more broadly. These researcher‐driven methods are particularly useful at smaller museums or institutions with limited funding and personnel, or those without permanent staff dedicated to collection management. In such cases, integrating curation and digitization with the research project can reduce museum expenses in terms of personnel costs and additional grants required for digitization. Researcher‐driven digitization is also beneficial when the study involves digital analyses or measurements of specimen images.

Overall, the workflow and protocols were found to be effective for processing fossil leaf collections of up to several thousand specimens. Streamlining tasks and workspaces improved the speed at which specimens were being processed and increased the flow of data, while minimizing specimen handling. Simplifying the workflow therefore improved outcomes, even though it invariably limits options and removes some freedom from the process. Because the protocols were developed based on a specific project at one institution, it is expected that procedures would need to be altered for use in other institutions or for other types of studies and/or specimens. For example, additional field and reporting protocols would be necessary for specimens collected from public and foreign lands. Additionally, the methods developed here are most applicable to specimens that do not need extensive preparation work, are relatively two‐dimensional, and can be studied and imaged without histological or three‐dimensional techniques. In general, this paper advocates that research projects involving the collection of new specimens should be planned and carried out to facilitate eventual sharing and digital archiving of both specimen and project data.

## Appendix 1 Workflow protocols for quantitative description of fossil leaf floras.

**A. Field protocol**

The following protocols are intended to be repeated over several field expeditions, each one followed by processing of the collected specimens in the museum. There are essentially two phases of fieldwork. The first phase, the diversity survey, is for establishing an initial set of sampling sites and collecting the first samples of specimens (steps 1, 2, and 4). These are brought back to the museum to study and delineate taxa. After processing the initial collections as per the museum protocol, a morphotype guidebook is compiled for use during subsequent field trips. The second phase of fieldwork is for performing field censuses at each locality to increase sample sizes (steps 2–4), and for establishing and sampling additional sites if needed (steps 1–4).

1. *Establishing and recording site information:* Establish independent sampling sites (e.g., quarries) that span the deposit(s) of interest and assign a unique locality number (quarry number) to each location. It is recommended to start with a small number of sampling sites spaced across the deposit (the number and spacing depending on the extent and preservation of the deposit). Additional sites can be added later if needed based on diversity trajectories, spatial heterogeneity, and project goals (see Burnham et al., 1992; Burnham, 1993). Record relevant site data (e.g., GPS coordinates, sedimentary information, any descriptive information, size of sampling area) in a field notebook. Photograph the locality and, if needed, collect sediment samples. All applicable local, state, federal, or international rules and regulations regarding permits and collecting should be followed.
2. *Quantitative sampling:* At each locality, sample the exposure in as large of blocks as possible that enable exposing the fossils. This will depend on the specific rock matrix, but also keep in mind that blocks can be broken down further after initial census of surfaces to reveal more specimens. Lay out all specimens recovered (a tarp is helpful to prevent losing specimens). In preparation for field census (step 3), it can be helpful to arrange similar specimens into groups for identification and census counts. Total sampling at each site should follow a predefined sampling scheme (e.g., by quarry size, number of specimens, species saturation, etc.). During censuses, sampling of the deposit may need be repeated following steps 3 and 4 to reach appropriate sample sizes. If sampling for the diversity survey, all recovered specimens should be collected for further study and to serve as the initial voucher specimens for morphotypes (skip step 3 and proceed to step 4). Note that for the diversity survey, specimens can also be selectively collected (“cherry‐picking”) from across the deposit or from float specimens. These specimens supplement the quarry samples in order to capture more of the potential diversity, aid in morphotype descriptions, and serve as morphotype vouchers (see Johnson, 2002). These specimens, however, are not included in the quantitative census data.
3. *Census data collection:* Identify specimens using the morphotype guidebook and perform census counts of fossil specimens on each slab of rock, assigning each slab a unique Rock ID number (see Wing et al., 2012 for census methods for percent cover). Record census data by Rock ID in field census data books. For the Rock ID, it is recommended to use a letter‐number combination for each collector to prevent using the same number twice (i.e., each collector is assigned a prefix letter and then numbers rocks starting from 001). Label each rock (preferably the sidewall of the rock that is void of fossils) with the Rock ID and quarry number using a permanent marker. Any new morphotypes recovered should be described in the field and collected for further study at the museum. Rocks with specimens that cannot be confidently identified in the field, but that have good preservation, should be numbered and set aside for further study at the museum. These can still be censused in the field with the unidentified specimens designated as such, and updated later after further study.
4. *Fossil collection:* Determine which specimens will be collected (for vouchers, further study, etc). If area/specimen‐based census data have been collected, trim the rocks down to the relevant specimens, if possible, to reduce excess matrix. For rocks that were not censused, such as the initial collections for diversity surveys, assign and label with a RockID and locality information. Wrap fossils in appropriate materials (e.g., newspaper, toilet paper, cellophane) for transport to museum. For all other remaining large blocks, break down the rock further to expose any other fossils present and repeat steps 3 and 4 as necessary.

**B. Museum protocol**

**Transition:**

1. *Organizing specimens:* Unpack fossils, match parts and counterparts, and organize them into specimen boxes and drawers. At this stage, organizing specimens into drawers by quarry (locality) is likely the most beneficial for subsequent processing. Within each quarry, group “like” specimens together to aid with later identification and description. Record the Rock ID of specimens into a curatorial log* spreadsheet as specimens are being unpacked. The curatorial log will serve as the primary management tool for later processing and thus should be set up to accommodate all future needs.
2. *Bridging data from field to museum:* Enter all data from the field census data books into a census data* spreadsheet. Using the Rock IDs, reconcile the curatorial log with the census data to determine which specimens were identified and counted in the field. A culled set of census data can be printed using a small font size, and then cut into small pieces of paper to place with each rock. This provides a simple way to associate the field identifications with the specimens for use during later processing, and provides an analog record of the census data. Additionally, all locality information, such as GPS coordinates and site descriptions, should be entered into the museum's database. If supported, field notebooks should be also scanned and uploaded.
3. *Setting up the workspace:* Prior to processing the new collections, a workstation should be prepared that can accommodate the different curatorial, data collection, and digitization tasks. The workstation should include a large general workspace for placing and handling specimens/drawers, as well as small task stations lined up along the workbench for photography, labeling, small‐scale fossil preparation, and microscopy. Some equipment needed for fossil preparation may not be accommodated within the workstation, such as large or dust‐generating equipment, due to associated safety protocols and/or best practices given the facilities available. See Nelson et al. (2012) for explanation of common imaging set‐ups.

**Integrated processing pipeline:** The following steps should be carried out completely for all fossils in a drawer, or collecting site, before proceeding to the next. Processing of each drawer through the pipeline will vary based on the needs of specimens within each drawer. Rocks in which all specimens were confidently identified in the field can be processed to completion independently. Rocks with potential new morphotypes or specimens that needed identification may need to be processed in batches of similar specimens to facilitate the circumscription of taxa.

1. *Fossil preparation:* Using techniques appropriate for the matrix type and fossil, remove any overlying matrix from specimens to expose specimens as completely as possible. Trim rock as necessary to reduce size for better fit in drawers. If a rock needs to be censused for area‐based counts including empty matrix (e.g., percent cover as in Wing et al. [1993, 2012]), trim only after these counts are completed.
2. *Identifying/describing specimens:* Study and identify all specimens on a rock, referencing the field census data (see step 6 above) when applicable. Census identifications can also be double‐checked at this time. Write new descriptions* or update previous morphotype descriptions as needed. A basic approach for unidentified specimens, such as the initial collections made for the diversity survey or potential new types recovered during census, is to first group/sort similar specimens (step 5), study to assess variability, compare with previously known morphotypes, and then circumscribe morphotypes based on detailed description of leaf characteristics (see Johnson, 1989; Ellis et al., 2009). The descriptions and representative images of specimens are compiled into a “morphotype guidebook” that is used for identifying specimens during field censuses and museum processing. The morphotype guidebook can also include notes about important characteristics that help differentiate between similar morphotypes and is continually updated with new information as the study progresses.
3. *Census:* For rocks that were part of quantitative sampling from quarries, perform census counts if not completed in the field and add to the census data. For incompletely censused rocks, update the census data with any new identifications that were determined from additional study.
4. *Labeling:* Labeling of specimens should follow protocols of the museum. Generally, when multiple specimens appear on a rock, each specimen should be assigned a unique number, which is labeled on the rock matrix near the fossil specimen. This can be a museum catalog number or extension of the field Rock ID number, depending on institutional practices regarding cataloging of voucher and non‐voucher specimens. A small strip of white gesso is painted onto the sidewall of the rock to accommodate writing basic data such as the Rock ID and locality number. Smaller dots of gesso can be applied near the individual specimens, on either the sidewall or surface, to label the individual specimen catalog numbers. A specimen label should remain with each rock or specimen, providing the identifications and other supporting information according to museum standards.
5. *Specimen imaging:* Imaging of specimens should follow a predetermined plan based on storage abilities and intended outcome. Preferably, every specimen cataloged would be imaged. However, other strategies include photographing only type specimens or exemplar specimens (i.e., ones that are well preserved, fairly complete, show unique features, or are useful for some type of later digital analysis). A widely used method for photographing specimens uses the manufacturer software provided with the camera to enable live view shooting through an attached computer (see Nelson et al., 2012), which provides fine control of focus and lighting and allows the specimen images* to be saved directly to the computer. Photograph specimens individually, using overhead and incident lighting that best captures a specimen's overall features. Center specimens and fill the field of view, with the major axis parallel with the long axis of the image, and include a scale bar. Take additional close‐up images of important characteristics as needed. Record the image numbers on a photography tag that can be placed with the specimen to denote completion of imaging. Metadata can be added immediately to individual images (or batches of images when there is more than one photo per specimen) using Adobe Bridge (Adobe Systems, San Jose, California, USA). Important metadata for a specimen may include the specimen number, quarry/locality number, GPS coordinates, morphotype/species identification, photographer, and copyright information. Image processing, such as lighting adjustments, can be performed at this time if needed.
6. *Specimen data entry:* Enter specimen information into the curatorial log, expanding the number of rows as needed so that each specimen occupies a single row in the datasheet. Enter all supporting specimen data, as determined by the museum's protocols and database software (see *Data files). If the method of data entry for the museum's database requires entering data on a specimen‐by‐specimen basis into specialized software (as opposed to batch uploads of specimen data from spreadsheets), enter into the museum system at this time. Otherwise, it can be uploaded in batches from the curatorial log.
7. *Re‐organization:* (optional) For long‐term storage of a collection, specimens may be organized by locality or by morphotype/species. Additionally, it may be desirable to keep voucher specimens of morphotypes/species, which may come from multiple sites, separate from the bulk of the collection, which is maintained by locality. After processing is complete, place specimens in appropriate drawers for their long‐term storage, and record drawer location in the curatorial log.

**C. Data mobilization and digital data use**

1. *Digital measurements:* (optional) Depending on project goals, specimen images can be used for digital measurement of specimen traits (e.g., Royer et al., 2005, 2007), using software such as ImageJ (Abramoff et al., 2004) or Adobe Photoshop (Adobe Systems). Measurements generated should be maintained in a trait measurement* spreadsheet, and optionally added to the curatorial log and/or museum collection records.
2. *Making data accessible:* After completion of the research project, including publication, all project data should be made publicly available via established databases appropriate for each data type. Suggestions of online repositories are included below for each data file mentioned in this protocol. In general, project data include all raw and derivative files for census and trait data, and supporting files for the morphotype diversity (morphotype guidebook or other descriptions). Although project data can often be included as supplementary files of the research publication, it is recommended to submit data to permanent online data repositories, such as Dryad (http://datadryad.org), if possible. The public accessibility and choice of online database for digitized specimen records and images will in most cases be determined by existing institutional practices, whether hosted by the museum's online collections records and/or other collections databases such as iDigBio (https://www.idigbio.org), PaleoPortal (http://paleoportal.org), the Paleobiology Database (https://paleobiodb.org/), and Specify (http://specifysoftware.org/).

***Data files: Explanation, formats, and databasing**

1. *Census data:* Census data should be tracked with a spreadsheet recording specimen counts, using simple column/row formatting that is appropriate for easy analysis in statistical programs such as R (R Core Team, 2016). For example, columns used here were Date, Quarry, Collector, RockID, Morphotype, No.Specimens, and No.Increments. Each row represents the unit of sampling; here, it is each morphotype on each rock. Save as a tab‐delimited text file or comma‐separated values file (.txt or .csv), which can be uploaded to Dryad (http://datadryad.org) upon completion of the project. Analyses of the census data may generate other derivative files, such as species occurrence or abundance matrices.
2. *Curatorial log:* This is a researcher‐generated spreadsheet managing all museum specimen data plus any additional information as determined by the needs of the research project. It should be organized with specimens as rows and data fields as columns (note that during the transition phase, each Rock ID is entered as a row, and additional rows are added during processing to accommodate multiple specimens per rock). This spreadsheet should be catered to the museum's collection management database/software and contain all required data fields. For example, if the museum's method of data entry for new collection records is a batch‐upload from an existing spreadsheet or form, the organization of the curatorial log should mimic that format for easy transfer of information. By completion of the project, all specimen data should be transferred, or entered, into the museum's system and made publicly accessible via the museum's online collections records and/or other online collections databases such as iDigBio (https://www.idigbio.org), PaleoPortal (http://paleoportal.org), or Specify (http://specifysoftware. org/).
3. *Specimen images:* Images should be saved in high resolution in camera RAW format for internal use, and in a more accessible format (such as .dng or .tiff) as determined by the requirements of the intended online repository. If possible, specimen images should be hosted by the museum's online database and/or linked to any online databases hosting the specimen records (e.g., iDigBio [https://www.idigbio.org]).
4. *Descriptions:* Descriptions should be managed using customized file(s) maintaining species/morphotype descriptions for all taxa in the assemblage. This includes the morphotype guidebook and/or any expanded version of the descriptions. Currently, there are no standard file types or database for managing morphotype information, especially that includes both the text descriptions and representative images. For angiosperm leaves, descriptive terminology and characters generally follow Ellis et al. (2009), for which a Microsoft Excel data entry template is available online. Until a public database is developed that hosts descriptive information (with or without systematic affinities known) along with images, this information may be best accommodated through publication of the research (in the manuscript text or supplementary information), or as a pdf uploaded to Dryad (http://datadryad.org).
5. *Trait measurements:* Record specimen trait data measured from digital images in a spreadsheet, with specimens as rows and traits as columns, formatted to facilitate analysis in statistical software. Save as a tab‐delimited text file or comma‐separated values file (.txt or .csv), which can be uploaded to a data repository, such as Dryad (http://datadryad.org), upon completion of project.
